# Psychomotor Functions and Interval Timing in Patients Receiving Intravenous Anesthesia for Endoscopic Procedures: The Pilot Study

**DOI:** 10.1100/2012/317897

**Published:** 2012-04-26

**Authors:** Włodzimierz Płotek, Marcin Cybulski, Anna Kluzik, Małgorzata Grześkowiak, Jacek Jelonek, Wojciech Switała, Jakub Janicki, Leon Drobnik

**Affiliations:** ^1^Department of Teaching Anaesthesiology and Intensive Therapy, Poznan University of Medical Sciences, 14 Marii Magdaleny St, 61-861 Poznan, Poland; ^2^Department of Clinical Psychology, Poznan University of Medical Sciences, 70 Bukowska St, 60-812 Poznan, Poland; ^3^Department of Anaesthesiology, Intensive Therapy and Pain Treatment, Poznan University of Medical Sciences, 49 Przybyszewskiego St, 60-355 Poznan, Poland; ^4^Department of Computer Science, Poznan University of Technology, 2 Piotrowo St, 60-965 Poznan, Poland; ^5^Faculty of Electronics and Telecommunications, Poznan University of Technology, 3A Piotrowo St, 60-965 Poznan, Poland

## Abstract

*Introduction*. The aim of this study was to evaluate two measures in a cognitive examination: psychomotor function and the perception of time (PT) in patients after intravenous anesthesia for endoscopic procedures. *Material and Methods*. We tested 23 anesthetized patients (Anesthesia Group, AG) and 17 not anesthetized patients (Control Group, CG). The Dufour Cross-Shaped Apparatus (DA) was used to assess quick reactions. Perception of time (PT) was measured for 1-, 2-, 5-, and 7-second intervals. The tests were performed before the anesthesia was administered and 1.5, 3, and 6 hours after the procedure was completed. *Results*. The intervals that were generated and the reproduced visual stimuli were shorter than the patterns. The reproduced 1- and 2-second auditory stimuli were longer than the patterns. The remaining reproduced auditory impulses were shorter than the patterns. *Conclusions*. In anesthetized patients, quick psychomotor reactions and the ability to time intervals are preserved 1.5 h and later after intravenous anesthesia for endoscopy.

## 1. Introduction

Many physiological systems in humans (e.g., hormonal, biochemical, and enzymatic) rely on one's timing to conform their level of activity to current environmental requirements, as governed by the suprachiasmatic nuclei [1]. Humans must also estimate multisecond intervals for everyday activities, such as handling a mobile phone and driving a car. This function can be expressed as interval timing (IT) (governed by the supplementary motor area and cingulated motor area) or chronometric counting (supported by Broca area, primary motor cortex and right cerebellum, and premotor circuit and cingulated motor area) [2]. These patterns are integrated by basal ganglia and transferred to the thalamus to generate behavioral responses [3]. Time studies can be conducted in prospective or retrospective paradigm. The first one is associated with cognitive structure, and the latter can be understood better in terms of knowledge. Main information on the time perception theories and methodology of its assessment has been provided by Taatgen et al. [4].

Quick reactions, within milliseconds, are supported by subcortical brain regions and the cerebellum and regulate one's ability to make fine, rapid movements [5]. 

We hypothesized that anesthesia effects impairments in psychomotor function and disturbances in time perception.

The purposes of this study were to evaluate the differences in the millisecond range of psychomotor reactions between intravenously anesthetized and nonanesthetized patients using the Dufour Cross-Shaped Apparatus (DA). Also, we examined the differences between groups in the change in perception of multisecond intervals using the interval timing apparatus (ITA).

## 2. Material and Methods

This study was performed with permission from the local bioethical committee (Bioethical Committee of our university, Permission No. 427/10, May 6, 2010). Patients gave written consent for participation in the study.

Between June and September 2010, 40 patients, hospitalized in the clinics of our university, were enrolled in the study; 23 (19 women and 4 men) who underwent a colonoscopy under intravenous anesthesia constituted the Anesthesia Group (AG), and 17 patients (6 women and 11 men) who did not receive such treatments formed the Control Group (CG). The information about basic gastrointestinal disorders of the participants was collected. The participants of both groups were selected randomly from the list of hospitalized patients and were offered the participation in the study. On the day prior to the colonoscopy under anesthesia, patients in both groups were asked to participate in the study and gave their informed consent. To exclude those with preexisting cognitive disturbances and depression, a screen was performed using the Mini-Mental State Examination (MMSE ≥ 24 pts) and Sense of Coherence Meaningfulness Subscale (SOC-29 ≥ 35 pts) [6–8].

Patients were clinically examined by a physician, and additional laboratory blood asseys were made with regard to dehydration, ion imbalances (potassium and sodium), severe anemia (Hb < 10 mg/dL), and thyroid dysfunction; the presence of any of these pathologies was a criterion for exclusion as the metabolic disturbances may be the causes of cognitive decline. On the day of the procedure, participants in both groups were tested psychologically using the Dufour DA to assess millisecond psychomotor reactions and the ITA to evaluate their perception of multisecond intervals (initial assessment); the tests are described below. The AG participants were premedicated with oral midazolam 0.1–0.15 mg/kg and transferred to the operating room. 

AG patients were anesthetized using intravenous anesthetics. In patients, propofol (Plofed 1%, WZF Polfa) 1-2 mg/kg was used. The drug provides 4-5-minute duration of anesthesia after single injection. Analgesic doses of fentanil (Fentanyl, WZF Polfa) 1-2 *μ*g/kg iv was used. During the anesthesia, vital signs were monitored. Vital signs were recorded, and no adverse events were observed. 1000 mL Sterofundin (Braun) was infused continuously to prevent dehydration. The duration of anesthesia was noted from the moment of the loss of eyelash reflex to spontaneous opening eyes and correct answer to a question about the patient's name. No patient suffered from postoperative nausea nor vomiting.

Measurements were repeated 1.5, 3, and 6 hours after the anesthesia wore off (when patients were fully conscious, oriented to time and place with stable respiration and Sat O_2_>90% and circulation: RR and HR ± 20% from baseline). The CG patients were tested at similar times. The results were discussed individually with the participants.

During the study, we administered the following tests.

For the Initial Screen:

The MMSE is a well-known test that is used to diagnose dementia early (cognitive disturbance was stated when MMSE ≥ 24 pts).

The SOC-29 comprises 8 questions that reveal depression (depression was stated when SOC-29 Meaningfulness Subscale ≤ 34 pts).

**Figure 1:**
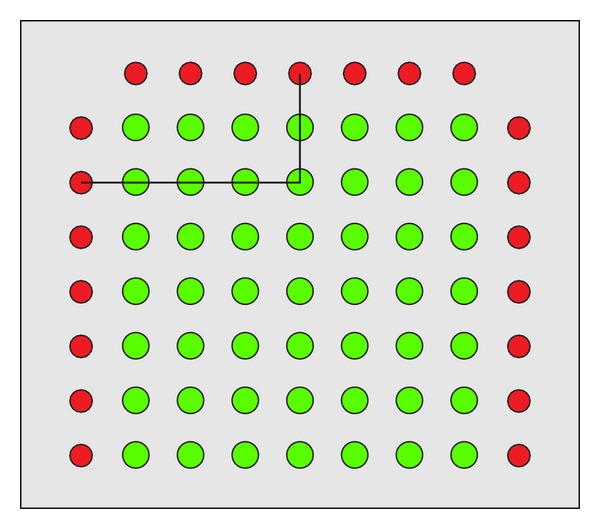
Front panel of Dufour Cross-Shaped Apparatus with marked operating rule. The patient's task is to press the green button that is marked by two lit red lights.

Psychomotor Evaluation and Perception of Time:

The Dufour Cross-Shaped Apparatus (DA)—model ATB/AK 2.0—is a diagnostic tool that tests visual and motor coordination and concentration used by traffic and transportation psychologists in Poland to diagnose drivers' cognitive functions [9]. The device (front panel) consists of 49 green buttons, surrounded by red-light-emitting stimuli. The red-light-emitting stimuli are placed on either side (for use by right- and left-handed persons) and on the top of the panel. The patient must press the green button that corresponds to the 2 red lamps from horizontal and vertical lines that are lit at the same time, testing their reaction to the light stimuli. The result is the total time of reaction to the set of the alternating 49 stimuli. The result is recorded in internal memory enabling their review on a display (back panel) (Figure 1).

**Figure 2:**
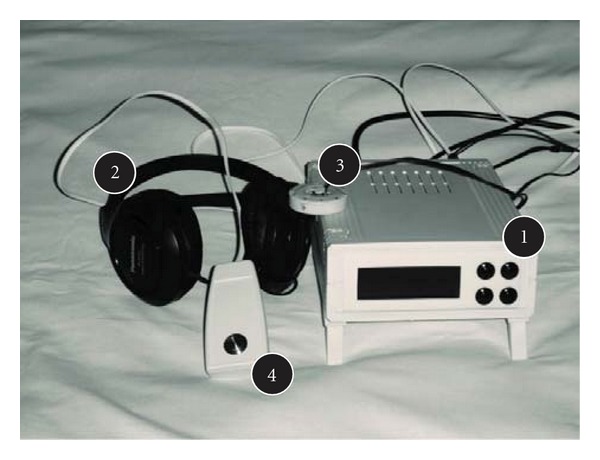
interval timing apparatus. The equipment consists of the main device (1), headphones (2), a laser projector presenting a contour image of an eye on the screen (3), and a controller (4). By pressing the button on the controller, the participant reproduces and produces intervals of time.

**Figure 3:**
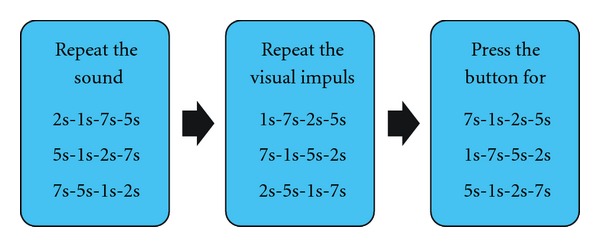
The interval timing assessment. The test consists of three parts: two on the reproduction of the given intervals, performed three times (“Repeat the sound,” “Repeat the visual impuls”), and one that is is based on interval production with no feedback (“Press the button for …). The participant is asked to repeat the durations of the sound from the headphones; the next step is to repeat the durations of the picture presented on the screen; finally, the patient presses the button on the controller for specific intervals of time. 1 s, 2 s 5 s, 7 s durations of the auditory or visual intervals presented or reproduced randomly.

The measurement of IT was performed according to the paradigm of interval reproduction and production described by Zakay [10]. The interval timing apparatus (ITA) is a device that was developed by scientists at the Poznan University of Technology (Figure 2). It generates auditory and visual impulses that are 1, 2, 5, and 7 seconds long, presented randomly in 3 trials for each modality. The auditory stimuli are sent through headphones, and visual stimuli are shown on a screen as an emotionally irrelevant picture of a contoured red eye. These procedures are standardized to exclude the confounders' impact. The test subject is asked to generate the same intervals 3 times without any prompt (Figure 3). The mean result of the 3 trials and its duration judgment ratio (DJR)—the duration that is experienced is divided by real duration of the stimuli—were calculated and analyzed.

### 2.1. Statistical Analysis

Statistical analysis was performed using PASW Statistics v.19 (2011) for Windows. The demographic data of the participants and DA and DJR results were expressed as mean ± standard deviation (SD). The differences between groups and in changes in DA and ITA performance within the tested groups were analyzed by Wilcoxon test; *P* < 0.05 was considered to be significant.

## 3. Results

The patients of the AG and CG were diagnosed with similar gastrointestinal disorders (AG: ulcerative colitis 6, Crohn's disease 6, chronic constipation 2, colon polyposis 2, diagnostic procedures 7; CG: ulcerative colitis 3, Crohn's disease 4, colon diverticulosis 1, diagnostic procedures 9). The education level between groups was comparable (*P* > 0.05): seven (AG: 5, CG: 2) received primary education, 22 (AG: 9, CG: 13) received secondary education, and 11 subjects (AG: 9, CG: 2) received higher education.

The demographics of the study participants are presented in Table 1. There were no statistically significant differences in demographics or cognitive screen results between groups (*P* > 0.05). 

The results on DA are presented in Table 2 (Table 2). No statistically significant differences were noted between groups with regard to performance on the all performances of DA (Wilcoxon test *P* > 0.05). Then, we analyzed the groups separately. By Wilcoxon test, within the AG, the performance on the DA 3 h after anesthesia was better compared with 1.5 h after the procedure, and the results after 6 h were better than 3 h after the procedure (*P* = 0.005 and 0.004, resp.). In the CG, better results on the DA were observed for 1.5 h versus initial assessment, 3 h versus 1.5 h and 6 h versus 3 h (*P* = 0.001, 0.001, and 0.001, resp.).

Table 3 presents the reproduction of the auditory stimuli in the groups. All of the reproduced 1-second and most 2-second auditory stimuli were longer than the patterns in both groups. Also, most of the reproduced 5-second and 7-second auditory stimuli were shorter than the patterns in both groups (Table 3).

By Wilcoxon test, single relationships were observed between the 1-second and 7-second intervals performed by the groups 3 h after anesthesia (*P* = 0.019 and 0.035, resp.). No other relationships were noted (Wilcoxon test; *P* > 0.05).

Most visual stimuli were reproduced as shorter segments in comparison with the patterns by both groups (Table 4). There were no differences between groups (Wilcoxon test; *P* > 0.05).

Most reproduced 1-, 2-, 5-, and 7-second intervals were shorter than the actual durations (Table 5).

There were no differences between groups in aspect of the time intervals produced with no initial pattern preoperatively and 1.5 h, 3 h, and 6 h after anesthesia (Wilcoxon test; *P* > 0.05).

## 4. Discussion

Many aspects of human cognition have been described with regard to anesthesiology [11, 12]. Impairments in cognition after anesthesia are an important consideration of perioperative medicine in major and minor surgical operations. During our study, the anesthetists applied the general intravenous anesthesia, which is associated with the application of opioid and anesthetic in accordance to good general practice. The anesthetists treated pain and abolished the consciousness in a controlled manner, thus providing comfort to the treated patients. 

The principal reasons that prompted us to select patients receiving the intravenous anesthesia were the growing number of 1-day surgical procedures and road traffic safety. Many of the short operations are performed as 1-day surgeries. The retention of intact psychomotor abilities after anesthetic administration is a demanding goal. Previous studies have used the Romberg test and static and dynamic posturographies to examine so-called street fitness—although the former is not accurate and the latter have not been used widely due to limited availability [13].

In this study, we used various tools of psychological testing. The tests (MMSE, SOC-29) are well described in the literature and are comprehensive for native Polish speakers [6, 7, 14]. They help to exclude subjects who suffer from pre-existing dementia and depression. The DA is a device that was developed according to the best practices for licensing professional drivers in Poland. The usefulness of this device in psychomotor testing has been evaluated [9]. The ITA is a device that was invented and produced by scientists at Poznan University of Medical Sciences and Poznan University of Technology to analyze the reproduction and production of time intervals, which have been the recognized paradigms of testing perception of time [10]. 

In this study, we wanted to determine how patients coped with time after anesthesia on short (analyzed by DA) and long scales (assessed by ITA). 

The DA results did not differ between groups, which confirms the good psychomotor status of the anesthetized subjects. Dressler et al. found that psychomotor function deteriorated up to 90 min after propofol anesthesia was administered, as assessed by the Short Performance Test, although memory impairments persisted for 24 hours [14].

Our study examined the recovery of psychomotor functions at a very early stage after anesthesia. There were no differences between groups 1.5 hours after intravenous anesthesia. These results on the DA are consistent with Riphaus et al., who did not observe any problems in psychomotor function, using the number connection test and a driving simulator, 2 hours after propofol administration, thus arguing against the strict regulations that forbid vehicle use 24 hours after anesthesia [15]. Our findings are also notable, because DA is widely used to test professional drivers in Poland [9]. The studies that have focused on different cognitive functions have used the Wechsler Adult Intelligence Scale, Emotional Stroop Test, and California Verbal Learning Test and are time-consuming and difficult in testing [16, 17]. The advantage of DA is its simplicity. Besides, the psychomotor function after anesthesia for one-day surgery is of extreme importance for the patient.

Because the perception of time encompasses elements of psychomotor reactions and working memory, the ITA results in both groups indicate that impairment relating to the elements of working memory engaged in ITA are not affected. Notably, anesthetized patients did not experience the “practice effect” on the DA test soon after the procedure. The possible experience acquisition is an undesired condition in repeated psychological testing but can be considered an aspect of human cognitive function. Collie et al. concluded that this effect was most robust when the test was applied for the first and second times and declines on subsequent applications [18]. In our study, results on the DA improved in the CG, but those of the anesthetized patients were unchanged soon after the anesthesia. The process of anesthesia disrupted the “practice effect.” It means that each next test performance could not be improved. This may be discussed as a sign of the cognitive disturbance despite the nonsignificant differences in the DA occurring between the tested groups. 

We were extremely interested in the aspect of timing, especially considering that Baldauf et al. observed that time production was a valid indicator of cognitive involvement in simulated driving [19]. The reproduced intervals and visual stimuli were shorter compared with the actual items. Only 1- and 2-second auditory impulses were perceived as longer, regardless of group. Longer auditory impulses were considered to be shorter. These results are partially consistent with Droit-Volet et al., who observed longer produced auditory stimuli (200–800 ms) compared with the exposure to visual impulses. The authors suggested that the modality of the signal prompts differences in the pacemaker speed of the internal clock [20]. Disparate modalities are performed by different regions of the brain, which can also affect the final production of time. 

In our study, we found no significant relationships in the groups between time reproduction and production. Likely, the impairments on ITA had withdrawn before the first postanesthetic examination, and we were unable to detect any differences. Everyday, anesthesiological practice proves how useful short-acting anesthetics are. The number of participants was a limitation of this study. Examination on a larger scale might have given us better insights into the problem of psychomotor functions and perception of time. Future studies should develop and evaluate objective measuring devices that combine various methods of screening patients objectively after anesthesia.

## 5. Conclusions

Anesthetized patients preserved quick psychomotor reactions tested by DA 1.5 hours after intravenous anesthesia for endoscopy, although the “practice effect” on this test is disrupted. They also preserved their ability to time intervals at this time point. The 1- and 2-second auditory stimuli that they produced were longer, and the reproduced 5- and 7- second impulses and all visual impulses were shorter. 

## Figures and Tables

**Table 1:** Demographics of the patients.

Parameter	Anesthesia Group, *n* = 23	Control Group, *n* = 17
	Demographic data	
Age (years)	42.56 (16.86)	38 (16.51)
Height (cm)	164.65 (9.97)	168.94 (11.04)
Body mass (kg)	64.43 (18.20)	63.06 (12.59)
BMI	23.63 (5.71)	22.30 (5.52)
MMSE (pts)	28.91 (1.53)	28.76 (1.44)
SOC-29 (pts)	43.61 (5.47)	42.17 (8.85)

Duration of anesthesia in minutes	36.74 (12.76)	Anesthesia was not performed

Results presented as mean and standard deviation (SD).

**Table 2:** Results on the Dufour Cross-Shaped Apparatus.

	Initial assessment	1.5 h	3 h	6 h
Anesthesia Group, *n* = 23	74.32 (18.61)	66.91 (35.18)	63.43 (24.76)	58.52 (30.57)
Control Group, *n* = 17	63.12 (32.78)	49.41 (29.81)	45.69 (25.97)	48.59 (25.22)

Results presented in seconds as mean and standard deviation (SD).

**Table 3:** duration judgment ratio (DJR) of the auditory stimuli.

	Initial assessment	1.5 h	3 h	6 h
Anesthesia Group's DJR (three consecutive trials), *n* = 23				
1 s	1.07; 1.03; 1.07	1.11; 1.20; 1.15	1.05; 1.09; 1.20	1.01; 1.09; 1.14
2 s	0.95; 0.91; 1.01	1.07; 1.09; 1.04	1.01; 1.04; 1.07	1.12; 1.11; 1.17
5 s	0.90; 0.85; 0.86	0.96; 0.92; 0.87	0.95; 0.94; 0.98	0.98; 0.99; 0.98
7 s	0.83; 0.89; 0.91	0.87; 0.96; 0.94	0.94; 0.92; 0.95	0.95; 0.99; 1.00

Control Group's DJR (three consecutive trials), *n* = 17				
1 s	1.19; 1.03; 1.00	1.21; 1.07; 1.28	1.24; 1.05; 1.19	1.13; 1.08; 1.12
2 s	0.96; 0.97; 1.00	1.06; 0.95; 1.07	1.00; 1.05; 1.05	1.00; 1.02; 1.02
5 s	0.81; 0.85; 0.85	0.90; 0.96; 0.96	0.96; 0.95; 0.97	0.93; 0.98; 0.97
7 s	0.91; 0.89; 0.87	0.93; 0.95; 0.97	1.02; 0.94; 0.96	0.96; 0.96; 0.99

Duration judgment ratio (DJR)—the duration that is experienced is divided by duration of the stimuli that are presented.

**Table 4:** Duration judgment ratio (DJR) of the visual stimuli.

	Initial assessment	1.5 h	3 h	6 h
Anesthesia Group's DJR (three consecutive trials), *n* = 23				
1 s	0.84; 0.93; 0.95	0.99; 1.00; 1.06	0.90; 0.99; 1.01	0.99; 0.96; 0.98
2 s	0.93; 0.84; 0.90	0.92; 0.86; 0.96	0.91; 0.84; 0.91	0.94; 0.88; 0.85
5 s	0.97; 0.91; 0.94	0.94; 0.93; 0.94	0.96; 0.95; 0.96	0.91; 0.92; 0.97
7 s	0.91; 1.00; 0.93	0.96; 0.93; 0.93	0.95; 0.94; 0.96	0.95; 0.94; 0.93

Control Group's DJR (three consecutive trials), *n* = 17				
1 s	0.88; 0.85; 0.90	0.93; 0.99; 1.03	0.86; 0.88; 0.83	0.81; 0.87; 0.92
2 s	0.87; 0.85; 0.90	0.92; 0.92; 0.91	0.94; 0.96; 0.91	0.99; 0.95; 0.96
5 s	0.83; 0.85; 0.84	0.83; 0.89; 0.93	0.87; 0.86; 0.92	0.91; 0.90; 0.95
7 s	0.87; 0.85; 0.81	0.90; 0.89; 0.90	0.88; 0.89; 0.90	0.89; 0.95; 0.92

Duration judgment ratio (DJR)—the duration that is experienced is divided by duration of the stimuli that are presented.

**Table 5:** Duration judgment ratio (DJR) of the time intervals produced with no initial pattern and 1.5 h, 3 h, 6 h after anesthesia.

	Initial assessment	1.5 h	3 h	6 h
Anesthesia Group's DJR (three consecutive trials), *n* = 23				
1 s	1.11; 0.96; 0.94	1.05; 1.08; 1.06	0.80; 0.82; 0.89	0.68; 0.89; 0.83
2 s	0.92; 0.91; 0.79	0.93; 1.07; 0.89	0.85; 0.88; 0.85	0.75; 0.83; 0.88
5 s	0.81; 0.80; 0.79	0.94; 0.94; 0.95	0.79; 0.85; 0.91	0.84; 0.92; 0.97
7 s	0.82; 0.79; 0.80	0.82; 0.90; 0.94	0.84; 0.89; 0.89	0.88; 090; 0.97

Control Group's DJR (three consecutive trials), *n* = 17				
1 s	0.71; 0.78; 0.84	0.71; 0.86; 0.88	0.74; 0.85; 0.95	0.76; 0.90; 0.86
2 s	0.63; 0.72; 0.76	0.76; 0.81; 0.81	0.73; 0.82; 0.80	0.71; 0.77; 0.78
5 s	0.73; 0.70; 0.74	0.74; 0.78; 0.78	0.80; 0.88; 0.85	0.78; 0.84; 0.86
7 s	0.69; 0.75; 0.73	0.82; 0.79; 0.82	0.84; 0.87; 0.81	0.84; 0.84; 0.90

Duration judgment ratio (DJR)—the duration that is experienced is divided by duration of the stimuli that are presented.
